# Anti-Cryptosporidial Drug-Discovery Challenges and Existing Therapeutic Avenues: A “One-Health” Concern

**DOI:** 10.3390/life14010080

**Published:** 2024-01-03

**Authors:** Munwar Ali, Chang Xu, Shah Nawaz, Ahmed Ezzat Ahmed, Qazal Hina, Kun Li

**Affiliations:** 1 Institute of Traditional Chinese Veterinary Medicine, College of Veterinary Medicine, Nanjing Agricultural University, Nanjing 210095, China; drmunwarali06@gmail.com (M.A.); 19552693850@163.com (C.X.); 2 MOE Joint International Research Laboratory of Animal Health and Food Safety, College of Veterinary Medicine, Nanjing Agricultural University, Nanjing 210095, China; 3 College of Veterinary Medicine, Huazhong Agricultural University, Wuhan 430070, China; malikshahnawaz786@gmail.com; 4 Biology Department, College of Science, King Khalid University, Abha 61413, Saudi Arabia; aabdelrahman@kku.edu.sa; 5 Department of Animal Nutrition, University of Veterinary and Animal Sciences, Lahore 54000, Pakistan; qazalhina5@gmail.com

**Keywords:** anti-cryptosporidial drugs, challenges, alternatives, ethnoveterinary, one health

## Abstract

Cryptosporidiosis is the leading cause of life-threatening diarrheal infection, especially in infants. Oocysts contaminate the environment, and also, being a zoonotic disease, cryptosporidiosis is a threat to One Health. Nitazoxanide is the only FDA-approved drug, effective only in immunocompetent adults, and is not safe for infants. The absence of mitochondria and apicoplast, the presence of an electron-dense band (ED band), hindrances in its genetic and phenotypic manipulations, and its unique position inside the host cell are some challenges to the anti-cryptosporidial drug-discovery process. However, many compounds, including herbal products, have shown efficacy against *Cryptosporidium* during *in vitro* and *in vivo* trials. Still, the “drug of choice” against this protozoan parasite, especially in immunocompromised individuals and infants, has not yet been explored. The One-Health approach addresses this issue, focusing on the intersection of animal, human, and environmental health. The objective of this review is to provide knowledge about novel anti-cryptosporidial drug targets, available treatment options with associated limitations, and possible future shifts toward natural products to treat cryptosporidiosis. The current review is organized to address the treatment and prevention of cryptosporidiosis. An anti-cryptosporidial drug that is effective in immunocompromised individuals and infants is a necessity of our time.

## 1. Introduction

Diarrheal infections are the leading cause of mortality among infants [1]. The apicomplexan parasite *Cryptosporidium* remains one of the major causes of lethal diarrheal infection [2]. It was recognized as a human health hazard during the AIDS (acquired immunodeficiency syndrome) epidemic of the 1980s [3], and cryptosporidiosis was included in the World Health Organization’s Neglected Diseases Initiative until 2004 [4]. The Global Enteric Multicenter Study (GEMS) reported that cryptosporidiosis ranks among the top causes of life-threatening diarrhea in children, especially in developing parts of the world, such as Asia and Africa [2,5]. More than 600 outbreaks were recorded in the US and England and Wales from 2009 to 2017 due to this protozoal disease [6,7]. In an outbreak in Sweden, about 50,000 sick-leave days were taken following waterborne *Cryptosporidium* infection, and 45% of 60,000 residents were ill [8]. During a waterborne epidemic in Ireland, 120,432 people were affected, costing around EUR 19 million [9]. *Cryptosporidium* is a serious health hazard, accounting for more than 8 million cases of foodborne illness annually [10].

It is a highly prevalent parasite because of its extensive host range, a greater number of infectious oocysts shedding from patients, transmission through water sources, and low infectious dose [11]. More than 15 species of *Cryptosporidium* are known to cause human infection, but two of them account for almost 90% of all cases: *C. hominis* (up to 80%) and *C. parvum* (up to 10%) [12]. The transmission route is the fecal–oral route [13]. Oocysts, the infectious stage of the parasite, are 4–6 μm in diameter, and due to the rigid, waxy wall made up of fatty tissue and glycoproteins, the parasite can survive in temperature extremes ranging from −22 °C to 60 °C, making it resistant to various disinfectants like chlorine and therefore difficult to control on livestock farms, in water supplies, in swimming pools, etc. [10,14]. The ED band of *Cryptosporidium* is not only involved in acquiring nutrients from the host-cell cytoplasm but also acts as an additional barrier to the delivery of systemic drugs to the parasite [15].

Cryptosporidiosis is a disease of zoonotic importance [13]. *Cryptosporidium parvum* can infect a variety of vertebrates, including humans and other animals, on a large scale, causing damage to the intestinal epithelium, triggering severe watery diarrhea, dehydration, and malabsorption syndrome in the affected individuals [16,17]. Diarrhea usually starts between 2 and 10 days after oocyst ingestion and leads to inflammatory changes in the GIT (gastrointestinal tract), especially the small intestine [18]. Also, a strong relationship between cryptosporidiosis and human colon cancer has been found [19]. Factors like parasite species, immune status, age, and type of host affect the pathogenicity of *Cryptosporidium* [20], as underfed children (under age 5), young children (0 to 24 months), and immunocompromised individuals are most likely to be affected by cryptosporidiosis [21]. It is an opportunistic infection in AIDS patients and people who have undergone transplant surgery or are suffering from tuberculosis (TB) [17]. *Cryptosporidium* may damage the respiratory tract, especially in patients with HIV (human immunodeficiency virus) [22,23].

Cryptosporidial oocysts contaminate the environment globally and are the biggest threat to the water industry [24]; in this environment, transmission between humans and/or animals occurs [25]. The prevalence of cryptosporidiosis is predicted to increase by up to 70% by 2050 in some parts of the world due to urbanization and climate change [26], so the prevention and treatment of this parasitic infection is a cause for concern, particularly in people with compromised immunity and in children. 

*Cryptosporidium* mostly affects cattle, water buffalo, camel, horse, sheep, goat, poultry, rabbit, pig, donkey, deer, wild mammals, and fish [27,28,29,30,31]. The prevalence of *Cryptosporidium* spp. infection was found to be 28.52% in cattle, 18% in buffalo calves, between 27.8 and 60.4% in pigs, 52.7% in dogs, and 29.4% in cats [32]. Recently, *C. parvum*’s presence in yaks has been reported in China, which may prove to be a serious food-safety threat, ultimately affecting public health [33]. Before weaning, *C. parvum* is the most common intestinal pathogen in calves [34]. One infected calf can shed up to 1 × 10^8^ oocysts in feces, posing a risk to other susceptible hosts [35]. Losses due to this disease in the cattle industry include calf mortality, diagnosis expense, medication and supportive care, and increased market age [36]. The global load of cryptosporidiosis in animal dung is 3.2 × 10 ^23^ oocysts per year, to which cattle is a major contributor [37].

Cryptosporidiosis is a global health concern, but treatment options are still suboptimal. The repurposing of a large number of medicines, including spiramycin, clofazimine, azithromycin, rifamycin, paromomycin, and HIV protease inhibitors, has been used in an attempt to treat PLWHA (people living with HIV/AIDS) and also suffering from cryptosporidiosis, but sufficient clinical success has not yet been achieved [38]; also, parenteral anti-cryptosporidial drugs are still not being focused appropriately [39]. To formulate a targeted drug for cryptosporidiosis, the essential steps are (i) to establish methods to identify anti-cryptosporidial compounds with different modes of action, and (ii) to separate these compounds into different groups according to their *in vivo* efficacy. This will aid in the choosing of more efficacious compounds [40]. Regarding the discovery of anti-cryptosporidial drugs, the processes in the “discovery phase” include *Cryptosporidium* biology, metabolism, the mechanism of *Cryptosporidium*-induced diarrhea, its interaction with host cells, the muco-adhesive properties of the drugs, and processes in the “developmental phase” including the use of different models like mouse, calf, gnotobiotic piglets, non-human primates, etc. to check the *in vivo* efficacy of different anti-cryptosporidial drugs. These drugs can be nonsystemic and systemic [41,42,43]. 

## 2. Methodology

**Figure 1:**
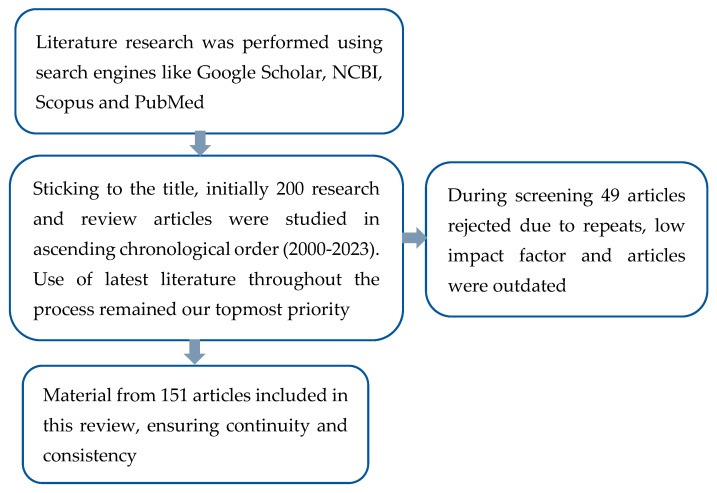
Methodology of the Review.

A rigorous search strategy was employed to retrieve articles from diverse databases. The literature search was conducted using a combination of keywords, terms, and Boolean operators regarding treatment options for *Cryptosporidium*, maximizing the scope of coverage and minimizing the risk of overlooking pertinent studies. The synthesis of findings involved a thematic analysis, categorizing and summarizing key themes and trends identified across the selected studies, providing a coherent narrative of the current state of knowledge in the field after intense selection to divert the attention of readers toward this multidisciplinary issue (Figure 1). 

Data from the literature was organized in this review to address the novel treatment options for cryptosporidium, especially focusing the immunocompromised patients and infants.

## 3. Cryptosporidiosis in the Perspective of “One Health”

**Figure 2:**
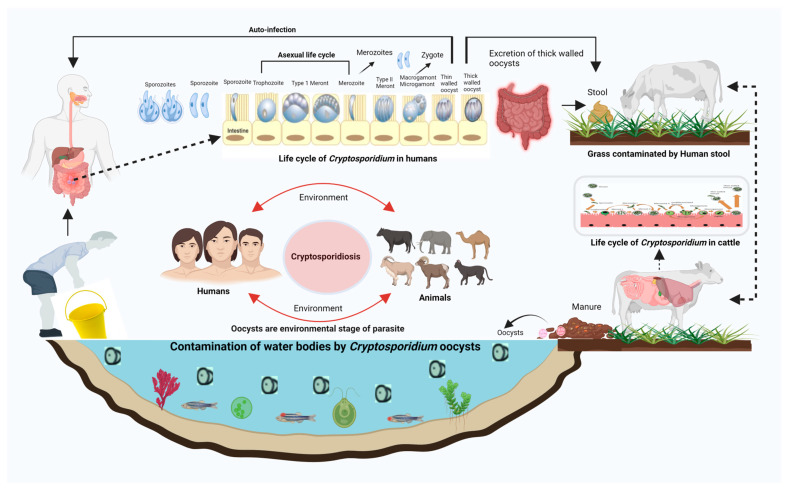
Cryptosporidiosis and One Health.

*Cryptosporidium* may be described from a One-Health perspective because it affects the health of humans, animals, and the environment at different levels [16]. An integrated, multidisciplinary, transboundary approach to detecting and differentiating this parasite in veterinary and public health will lead to source tracking, surveillance, data collection by experts, and the dissemination of information to the public. Developing new vaccines for animals and people to reduce disease burden is the basic “One-Health” goal. The application of relevant clinical *in vivo* models and novel *in vitro* trials will help to understand host–pathogen interactions and will make it possible to test the efficacy of new treatment options and vaccines. Novel methods to treat *Cryptosporidium*-contaminated livestock manure and human excrement will reduce oocysts’ environmental contamination and help protect water catchments. Knowledge exchange and education among professionals from all the sectors of One Health is mandatory to tackle such One-Health threats (Figure 2) [16]. 

Oocysts are the environmental stage of cryptosporidiosis. Oocysts excreted in the human stool contaminate the environment and vegetation and finally reach another host, such as cattle. After completing their life cycle in the intestine of the second host (e.g., cattle), they are excreted in the manure and once again contaminate areas of the environment, such as water bodies. This cycle continues among different components of One Health.

## 4. Hurdles in Developing the Anti-Cryptosporidial Drugs

There are two drug-discovery screens: (i) cell-based (phenotypic), and (ii) target-based, and each has its own merits and demerits [44]. *Cryptosporidium*, unlike other protozoan parasites, has eliminated organelles like apicoplast and mitochondrion. Also, the unique location of this parasite, separated from host-cell cytoplasm in the enterocytes, and its effect on the biliary duct pose some challenges to the pharmacokinetics of anti-cryptosporidial drugs [4]. The genetic manipulation of *Cryptosporidium* has been a major hurdle in developing vital translational research tools to develop drug targets in the *Cryptosporidium* [44]. However, presently, CRISPR/Cas9, a technology that enables gene editing, can be used to genetically modify the parasite to generate anti-cryptosporidial drug targets in the parasite [16]. Phenotypic screening of *Cryptosporidium* is also a challenge because, using the current *in vitro* techniques, it is not possible to capture all stages of the protozoan life cycle because the present assays do not support the continuous culturing of *Cryptosporidium* in the laboratory. Also, oocysts lose infectivity after 6–8 weeks of shedding in feces, so continuous culturing in animal models is necessary, which is a difficult task. However, *in vitro* culturing of the oocysts can be done using an HCT-8 (a human colorectal carcinoma cell line obtained from an adult male) organoid model [45], primary cultured enterocytes, and small-intestinal epithelial FHs74 cells (human fetal small-intestine cells) [46,47]. Different models are available for *C. parvum*. However, gnotobiotic piglets and immunocompromised gerbils are the only animal models available for *C. hominis* [44,48]. Limitations and the low availability of animal models are big issues. The piglet model has the advantage that infection of both *C. parvum* and *C. hominis* can be studied in it [41,42,43]. 

Anti-cryptosporidial drugs must easily cross at least three barriers, i.e., the host epithelial membrane, the parasitophorous vacuole membrane, and the parasite membrane [49]. Also, if broad-spectrum antibiotics are being used against *Cryptosporidium*, then it will definitely disturb the normal microflora of GIT. Importantly, the presence of severe diarrhea washes out the drug before its action, reducing its efficacy [50,51]. Therefore, compounds with high GIT remain, but low systemic availability is needed to achieve sufficient efficacy and safety margins to treat cryptosporidiosis. Also, the small intestine is the most important extrahepatic site for drug biotransformation by cytochrome P450-mediated phase-I drug metabolism, so these biotransformations should also be a matter of concern while preparing a drug candidate against cryptosporidiosis [52]. Also, the anti-cryptosporidial compound should remain intact in the intestinal mucosa, especially during lead-optimization efforts [49]. Regarding the availability of anti-cryptosporidial drugs, it is noteworthy that binding the drug with the plasma proteins reduces its potency. As multi-pathogen infections are expected, the drug should be effective after drug–drug interactions [53]. 

## 5. Anti-Cryptosporidial Drug Avenues with Their Target Sites, Effectiveness, and Limitations

Many anti-cryptosporidial drugs like nitazoxanide, clofazimine, BRD7929 (bicyclic azetidines), 5-fluoro-2′deoxyuridine (FDU), halofuginone, and other pharmacological candidates have been tested in controlled/uncontrolled clinical trials, open-label blind studies and case reports [54]. The following are some potent and common anti- cryptosporidial compounds with their respective merits/demerits.

Nitazoxanide (NTZ): NTZ is the only approved drug for cryptosporidiosis [22], but it is equal to placebo in immunocompromised patients and is only approved for children ages 1–11 years [55]. Thus, there is a clear need for improved drugs to treat cryptosporidiosis, especially in children and immunocompromised people such as those who have AIDS [40].

Clofazimine (CFZ): CFZ is an FDA-approved riminophenazine antibiotic against leprosy that shows strong anti-cryptosporidial activity in the mouse model [55]. Contrary to this, it was ineffective in a phase-2 human trial [38]. During the *in vitro* trial, CFZ inhibited *C. parvum* proliferation by 70% at every point of its asexual life cycle with EC99 (effective concentration 99) of CFZ = 30 nM, compared to the 5-fluoro-2′-deoxyuridine (*FDU*) = 100 nM; bumped-kinase inhibitor-1294 (BKI-1294) = 2 μM. Therefore, CFZ is comparatively more efficacious [55]. However, CFZ has limited oral bioavailability, so it should be encapsulated as a micronized suspension in a lipid wax base [55]. 

Glycolipopeptide Occidiofungin: More recently, a glycolipopeptide occidiofungin was found to be a potent drug against *C. parvum* with poor absorbability and GIT retention *in vitro*, with limited cytotoxicity. However, muco-adhesiveness should be a priority for orally administered drugs [56].

Pyrazolopyridine derivatives (KDU731): This is a potential lead compound, was initially found to be an anti-malarial drug, and is, presently, the most advanced compound in the anti-cryptosporidial drug pipeline [57]. It is proven to be effective in calves. Trials in humans for safety and pharmacological evaluations are still ongoing. Considering its pharmacokinetics, the selectivity index of KDU731 is >100 and shows a half-maximal effective concentration (EC50) of *C. parvum* (CPE* = 0.1 μM [57]. CPE* = cytopathic effect).

### Calcium-Dependent Protein Kinase (CDPK) Inhibitors

(i) Bumped-kinase inhibitor-1294 (BKI-1294): This inhibits calcium-dependent protein kinases (CDPKs) without affecting the host-cell activity because CDPK in *Cryptosporidium* are somewhat different from those found in mammalian cells, as their active site contains a glycine instead of bulkier gatekeeper residues that are found in mammalian CDPKs. Bumped-kinase inhibitors have proved efficacious in targeting these kinases in different animal models. However, their anti-human side effects are still unclear [58].
(ii) Imidazole-pyrimidine: This is another CDPK that has proved effective in treating cryptosporidiosis and has exhibited favorable safety and pharmacokinetics when used in a mouse model at a dose of 30 mg/kg daily. It is reported that the imidazole-pyrimidine compound inhibits CDPK-1 in *C. parvum*, with a 50% inhibitory concentration (IC50) of 2 nM. However, there is still some ambiguity regarding its exact mechanism of action [59].

Cyclosporine: This inhibits calcineurin’s phosphatase activity [60]; this compound can be tested for the treatment of cryptosporidiosis.

The anti-cryptosporidial effect of Compound 5 (cladosporin derivative) on lysyl-tRNA synthetase (KRS) [61], the effect of triacsin-C on acyl-CoA synthetase (ACS) [62], the influence of P131 (lead compound designed to be retained in the GIT) on inosine-5′-mono-phosphate dehydrogenase (IMPDH) [63], the effect of gossypol on lactate dehydrogenase (LDH) [64], the effect of vorinostat on histone deacetylase (HDAC) [65], and BRD7929 (bicyclic azetidine compound) effects on phenylalanyl tRNA synthetase (PheRS) [66] were checked by Vinayak et al. [66], and favorable results were shown by these compounds, targeting different enzymes. 

Compounds like valinomycin, mitomycin, dactinomycin, daunorubicin (the latter two derived from streptomyces), 3-deoxo-3beta-hydroxymexicanolide-16-enol ether (plant origin), tanshinone-II-A, baicalein, deoxysappanone-B 7,30-dimethyl ether acetate, dihydrogambogic acid, deacetylgedunin, cedrelone, deoxysappanone-B 7,40-dimethyl ether (Deox B 7,4), deacetoxy-7-oxogedunin, dihydrotanshinone-I, 2,3,40-trihydroxy-4-methoxybenzophenone, 3-deoxo-3beta-hydroxymexicanolide-16-enol ether (plant origin), and lovastatin (*Aspergillus terreus*) proved efficacious against cryptosporidiosis with anti-cryptosporidial EC50 values ranging from 0.122 µM to 3.940 µM. These compounds need further evaluation for their suitability in animal models [67]. Also, compounds such as 6-carboxamide benzoxaborole (AN7973, AN7973) [68], acetyl CoA binding protein (ACBP) inhibitor, ellagic acid (a natural compound), alisol-A, alisol-B, atropine sulfate, and bufotalin, are some anti-cryptosporidial compounds (Table 1). Atropine sulfate and bufotalin displayed excellent anti-cryptosporidial activity [69].

As there are no functional mitochondria in *C. parvum*, it depends upon glycolysis to fulfill its metabolic needs. Therefore, the glycolytic enzymes can be the target to inhibit the growth and survival of *Cryptosporidium* [70], as the significance of glycolytic enzymes like hexokinase (CpHK) and glucose-6-phosphate isomerase (CpGPI) make them options to treat the cryptosporidiosis [71,72].

*Cryptosporidium* enzymes like Phosphatidylinositol-4-OH kinases (PI(4)Ks), which play an essential role in intracellular signaling and trafficking [73], and another enzyme, Nucleoside Diphosphate Kinase (NDK), are required to synthesize nucleoside triphosphates, maybe anti-cryptosporidial drug targets. Castellanos-Gonzalez and colleagues developed single-stranded RNA (ssRNA) and the human enzyme Argonaute 2 (hAgo2) complex to silence *Cryptosporidium*’s targeted mRNA for NDK. This inhibits the NDK [74]. Similarly, thymidylate-synthetase dihydrofolate reductase (TS-DHFR) converts the tetrahydrofolate by dihydrofolate reductase to synthesize the genetic material [75]. Also, Cysteine Protease (N-methyl piperazine-Phe-homo Phe-vinyl sulfone phenyl (K11777) is its inhibitor) [76], and 3-hydroxy-3-methyl-glutaryl-coenzyme-A reductase (HMG-CoA reductase) [74,77] may be the targets to inhibit the growth of *Cryptosporidium*. 

Probiotics: The therapeutic effect of probiotics, such as *Enterococcus faecalis* CECT7121, on *C. parvum* infection could be attributed to competition for binding sites on the gut epithelium, the acidification of the medium induced by lactic acid bacteria [78], an increase in the number of IgA-producing cells, and increased production of IgM [79]. Similarly, *Cryptosporidium* infection in neonatal rats showed a trend where using probiotics during the infection led to more clearance of cryptosporidiosis; however, daily administration of *Lactobacillus casei*-containing mixtures in neonatal rats model did not remove the protozoan [80]. Further research is needed to check the anti-cryptosporidium effects of different probiotics. Some anti-cryptosporidium drugs are mentioned in Table 1.

**Table 1:** Anti-Cryptosporidium drugs and their mode of action.

Anti-Cryptosporidial Drugs	Mode of Action	General Characteristics	References
Nitazoxanide	Inhibits PFOR*	Less effective when there is dysfunctional Th1* immunity because CD4 and natural killer cells play a role against the parasite	[81,82]
BRD7929 (Bicyclic azetidines)	Inhibits parasite phenylalanyl tRNA synthetase	Kill diarrheal pathogen *Cryptosporidium*	[83]
Clofazimine (CFZ)	Inhibit the asexual phase of development	Tissue distribution is very high, leading to strong pigmentation in the skin	[55,84]
5-fluoro-2′deoxyuridine (FDU)	Inhibits *C. parvum* thymidine kinase, interferes with DNA synthesis	Inhibit *C. parvum* without any harm to HCT-8* host cells	[85]
Paromomycin	Inhibits protein synthesis by binding to the prokaryotic ribosomes of *Cryptosporidium*	Effective in the immunocompromised patients (AIDs) to treat the cryptosporidiosis	[86]
Halofuginone	Target cytoplasmic prolyl-tRNA synthetase in *Cryptosporidium*	Hepatotoxicity and GIT disturbances	[87,88]
Pyrazolopyridine (KDU731)	Phosphatidyl-inositol 4 kinase (PI4K) inhibitor	Active against both *C. parvum* and *C. hominis*, and also in immunocompromised mice	[57,83]
3,5-dipyridyl-triazole (NSC234945) and Dihydronaphthalenone (NSC252172)	CpPyK* and LDH* inhibitor inhibits the parasite’s glycolytic pathway and, hence, ATP generation	Efficacious in immunosuppressed mice at a dose of 10 mg/kg of body weight	[64,89]
6-carboxamide benzoxaborole (AN7973)	Arrest DNA synthesis inhibits thymidine EdU* into newly synthesized DNA for elongation of the DNA strand	Efficacious against both *C. hominis* as well as *C. parvum*, in immunosuppressed mice too	[40,83]
Alisol-A, B, Atropine sulfate, and bufotalin	Alisol-A, B, inhibit the endoplasmic reticulum Ca^2+^ ATPase Atropine sulfate blocks the parasite’s metabolic route	Low EC50 values, minimum cytotoxicity in human enteric cells (HCT-8).	[90,91,92,93]
ACBP inhibitors (Broxyquinoline, cloxyquin, cloxacillin sodium)	Inhibit *C. parvum* *in vitro* by targeting CpACBP1*	ACBP is a family of housekeeping proteins that play a role in controlling lipid metabolism	[94,95]
Pyrazolopyrimidines, 5-aminopyrazole-4-carboxamide, imidazole-pyrimidine	Inhibits *C. parvum* calcium-dependent protein kinase-1 (CDPK1)	Proved efficacious at a dose rate of 100 mg/kg in a mouse suffering from chronic cryptosporidiosis	[4,58,96]
Lead compound P131	Inosine monophosphate dehydrogenase (IMPDH) inhibitor	Stay longer in GIT	[63]
Vorinostat	Histone deacetylase (HDAC) inhibitor	Decreased the inflammatory mediators by up to 50%, effective against latently HIV*-infected T lymphocytes	[21,65,97,98]
Ellagic acid	Inhibits nucleoside diphosphate kinase-B (NDK)	Effective in infected HCT-8 cells with EC50 range in 15-30 μM	[99]
Halogeno-Thiazolides	Inhibit *Cryptosporidium* growth and oocysts shedding	Effective in immunosuppressed patients	[100]
Buparvaquone	Not well known	Reduces parasite growth without toxicity	[101,102]

(* = represent that the full form/explanation of this abbreviation is given in the footnote of table). PFOR (Pyruvate-ferredoxin oxidoreductase), HCT-8 cells (human colorectal carcinoma cell line obtained from adult male), EC50 (effective concentration-50), Th1(T-helper cells, EdU (5-ethynyl-2′-deoxyuridine), CpPyK (*C. parvum* pyruvate kinase), LDH (lactate dehydrogenase), ACBP (Acetyl CoA binding protein), CpACBP (*C. parvum* Acetyl CoA binding protein), HIV (Human Immunodeficiency Virus).

## 6. Ethnoveterinary Approach to Treat Cryptosporidiosis

The extensive use of antiparasitic chemical medicines increases the parasitic resistance to current treatments, inefficiency, toxicity, prolonged treatment duration, and cost [103]. Medicinal plants and natural products have been used for a long time due to their therapeutic effects. About 80% of the population in developing countries still uses herbal products and medicinal plants for different health problems, especially in animals [104]. Medicinal plants and their metabolites contain several organic compounds that have powerful applications as antiparasitic drugs to prevent and cure many parasitic infections [105].

The efficacy of NTZ using an immunosuppressed mice model was recorded at a dose rate of 100 mg/kg. At 10 *C. parvum* oocyst exposure, it proved ineffective. Therefore, ***Verbena officinalis*** (***V. officinalis***) was used to check its anti-cryptosporidial efficacy [106]. In immunosuppressed infected mice, *V. officinalis* and NTZ, when used in combination, showed novel results (87% efficacy), reducing the shed of oocysts in stools with minimum side effects [107]. Histopathological changes were minimal when this was used with the normal presence of goblet cells, and liver architecture was not disturbed (Table 2) [108].

***Moringa oleifera*** is another potent drug candidate. Its leaf extract (EMOLE) contains phenolic compounds like chlorogenic acid (CGA) and is known for its anti-inflammatory, antidiabetic, anti-cancer, and antiprotozoal effects. It is also effective in immunosuppressed individuals [109,110]. When used in concentrations like 10, 20, 30, and 40 mg/kg/day of CGA, even when *in vivo* administration of 100 mg/kg was given, no toxicity was observed [111,112]. In another study, treatment with EMOLE led to an 85.2% to 91% reduction in the oocyst excretion in accordance with similar studies [113] and may have improved the host immunity, too [114]. Therefore, it has anti-inflammatory properties (Table 2). Meanwhile, treatment with EMOLE decreased mucosal damage and improved immune response. Most villi were intact, comparable to NTZ-treated mice [115,116,117].

**Figure 3:**
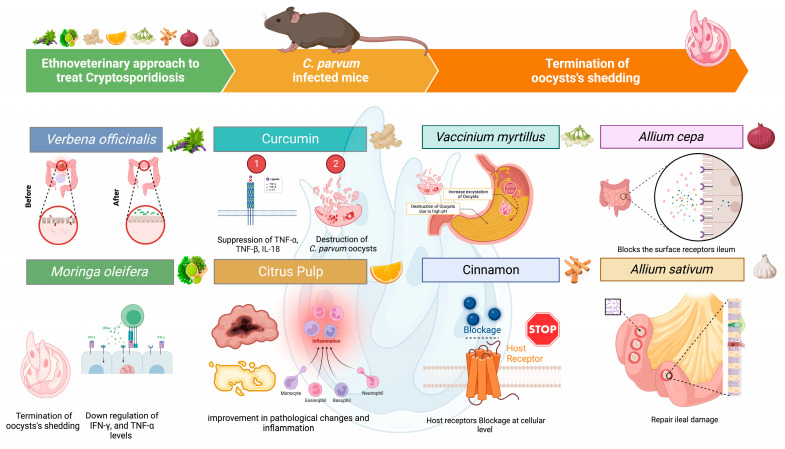
Use of the ethnoveterinary approach to treat cryptosporidiosis.

**Curcumin** (1,7-bis(4-hydroxy-3-methoxyphenyl)-1,6-heptadiene-3,5-dione) is commonly referred to as diferuloylmethane, an orange–yellow ingredient that is derived from ***Curcuma longa***(turmeric) [118]. Recent research indicated that curcumin is an effective compound against *C. parvum* infection [119,120]. In an experiment, when compared to the infected (10^6^ oocysts) untreated group, a 74.70% reduction in oocyst shedding was observed in the infected curcumin-treated group and 60.68% (*p* < 0.01) compared to the infected Paromomycin-treated group. After curcumin treatment, villi were elongated, epithelial damage was insignificant, there was no hyperplasia in some of the villi, and a renewal of the brush border in the ileum of the curcumin-treated group was observed. At excretion day 5, *Bacteroidetes* increased significantly (0.35%) in the group treated with curcumin. The population of *Firmicutes* and proteobacteria increased in the infected untreated group [121] (Figure 3). A benefit of curcumin is that it is non-toxic to the host and does not damage the kidney [122]. ***Citrus maxima*** and **pomegranate *(Punica granatum)*** peel against *C. parvum* in the immunosuppressed murine model was found to be effective, too [113] (Table 2).

Medicinal herbal products like curcumin, *Moringa oleifera* leaf extract, chlorogenic acid, onion, garlic, blueberries, pomegranate, cinnamon, citrus, and thyme extract and their metabolites contain several organic compounds that are powerful antiparasitic drugs used for the prevention and cure of cryptosporidiosis.

**Table 2:** Herbal products and their anti-Cryptosporidial effects.

Herbal Product	Active Ingredient	Mode of Action	Reference
*Curcuma longa* (Turmeric)	Curcumin	Increases the expression of IL-18* and IFN-γ*. It modulates the gut microbiome, increases lactobacilli, and enhances innate immune response by increasing the expression of IL-18* and IFN-γ*	[121]
*M. oleifera* leaf extract	Antioxidants Flavonoids, Alkaloids, Steroids, Tannins	EMOLE* and CGA* in *C. parvum* infected mice lead to the downregulation of IFN-γ, IL-6, IL-1β, and TNF-α* levels significantly	[123,124,125]
*Allium sativum* (Garlic) extract	Garlicin’s diallyl disulfide, Diallyl trisulfide	Disrupts mobility, nutrient absorption, and reproduction, modulates the activity of macrophages, inhibits TNF-α and releases cytokines and chemokines, and activation of NK cells	[126,127]
*Citrus* peel	Phenolic compounds	Interfere with ATP generation by uncoupling oxidative phosphorylation	[128,129,130]
*Thymus vulgaris* extract	Phenolic compounds	Thymus extract blocks the *Cryptosporidium* receptors of the intestinal mucosa. It protects essential enzymes from oxidative damage	[131,132]
*Verbena Officinalis* extract	Iridoids, Flavonoids, Phenolic acid	Anti-inflammatory and analgesic chemicals in the extract of *V. officinalis* extract. Less ileal damage after the treatment with *V. officinalis*	[133]
*Artemisia herba-alba* extract	Artemisinin	Inhibits *Cryptosporidium* penetration into enterocytes by blocking receptors on enterocytes, causing parasite calcium element disruption and leading to decreased parasite colonization	[134]
*Punica granatum* (Pomegranate) extract/peel	Polyphenols and Tannins	Eliminates oocyst shedding and reduces *C. parvum* trophozoites and lymphatic infiltration	[135]
*Olea europaea* L. extract	Oleuropein, luteolin-7-glucoside, Apigenin 7-7-glucoside	Compete for or block *Cryptosporidium* receptor sites on the ileal epithelium and reduce the *C. parvum* colonization	[136,137]
*Allium-cepa* (Onion)	Flavonoids, Alkaloids, Terpenoids	It blocks the surface receptors of the ileum for *C. parvum* and prevents oxidative stress	[138,139]
*Cinnamomum zeylanicum* (Cinnamon)	Phenolic compounds	Its extracts impair the integrity of the parasite membrane and neural signal transmission. Cinnamon oil blocks *Cryptosporidium* receptors on the enterocytes	[138,140]
*Vaccinium myrtillus* (Blueberries) extract	Polyphenolic compounds, Anthocyanins	Induces the spontaneous excystation of oocysts in both the stomach and intestine, exposing sporozoites to acidic pH, and antiglycative and antioxidant characteristics	[141]
*Mangifera indica* (Mangiferin) extract	Rutin, Epicatechin	Antioxidant and free radical-scavenging properties, reduces the *C. parvum* colonization	[142,143]

(* = represent that the full form/explanation of this abbreviation is given in the footnote of table). Interleukin-18 (IL-18), Interferon-γ (IFN-γ), *Moringa oleifera* leaf extract (EMOLE), chlorogenic acid (CGA), tumor necrosis factor-α (TNF-α).

## 7. Preventive Strategies

At present, there is no effective cryptosporidial vaccine for humans or livestock, and limited therapeutic options are available to treat this zoonotic infection. However, vaccines against diarrheal pathogens (e.g., *Escherichia coli*, rotavirus, coronavirus) may be effective for cryptosporidiosis in the dam and passive immunity transferred to calves via colostrum, thus preventing infection in calves in the early stages of life. Using recombinant CP23 (*Cryptosporidium* protein 23) and CP15 proteins to produce colostrum with high antibody titer, protecting neonatal calves against cryptosporidiosis is an optimistic initiative [53,144]. Disinfection with hydrogen peroxide and hydrated lime is good [145] to prevent cryptosporidiosis in calves [146,147]. Acidic pH and anaerobic digestion by mesophilic and thermophilic bacteria significantly inactivate oocysts [37]. A rapid gravity filtration system proved efficacious in removing oocysts from finished water [148]. The formulation of a drug as a muco-adhesive nanosuspension to compensate for the situation of severe diarrhea (which leads to the rapid excretion of the drug) and also as a muco-adhesive hydrogel will prolong retention time and contact time of the drug to a given pathogen, therefore improving its bioavailability [101]. For example, using muco-adhesive buparvaquone with chitosan as a polymer would reduce its dose, reducing adverse effects in treating intestinal cryptosporidiosis [101].

## 8. Future Research Perspective

The studies investigating the effectiveness of vaccines in reducing the occurrence of cryptosporidiosis and the shedding of oocysts into the environment, source tracking, epidemiological evaluation, and surveillance in veterinary and public health fields should be focused in the future. The control of *Cryptosporidium*, finding effective treatments to reduce the viability of cryptosporidial oocysts in excreta, discovering a novel anti-cryptosporidium drug that would be effective in both humans and animals, and the use of CRISPR/Cas9 (a technology that enables insertions, deletions, and other manipulations of genes of interest) to find anti-cryptosporidium drug targets [16] may be future research perspectives. Finding the permeability of different anti-cryptosporidial compounds, especially bumped-kinase inhibitors to small-intestinal cells, the capacity of the parasite to target the specific molecules of host cells, the exact mode of action of some anti-cryptosporidial compounds [149,150], and the relationships between gut microbiota and these protozoa in infected animals [151] may be future research interests.

## 9. Conclusions

Many compounds such as nitazoxanide, clofazimine, paromomycin, halofuginone, muco-adhesive buparvaquone, pyrazolopyridine (KDU731), vorinostat, halogeno-thiazolides, ellagic acid, and herbal products like curcumin, *Moringa oleifera* leaf extract, chlorogenic acid, onion, garlic, blueberries, pomegranate, cinnamon, citrus, thyme extract, etc. have shown efficacy against the *Cryptosporidium* in different experimental setups by targeting different enzymes, interfering with reproduction and the host–pathogen relationship, and blocking receptor sites for *Cryptosporidium.* However, a potent target drug that would be effective in immunocompromised individuals and infants has not yet been explored. This requires a global collaborative effort involving ecologists, pharmacologists, regulatory agencies, and product development partnerships.
